# S9-1 How to stimulate active ageing? Cross-national insights to improve recruitment, participation, fall prevention and scalability

**DOI:** 10.1093/eurpub/ckad133.043

**Published:** 2023-09-11

**Authors:** Chair(s):, Katja Borodulin, Jannique van Uffelen

**Affiliations:** Age Institute; KU Leuven

## Abstract

With increasing numbers of older persons in the world, new practices and programs are needed to support active aging. These new practices and programs should rely on research-based evidence, be tested in real-world settings, and also be both sustainable and scalable to the society.

In this symposium we aim to share practice-to-policy-related examples from four countries, where issues related to transition to retirement, recruitment challenges, and fall prevention are covered.

Cases rely on research-based knowledge, but concentrate on presenting results from practice, implementation and scaling up phases. Issues related to challenges and solutions in each case are shared and discussed.

